# The major histocompatibility complex (*Mhc*) class IIB region has greater genomic structural flexibility and diversity in the quail than the chicken

**DOI:** 10.1186/1471-2164-7-322

**Published:** 2006-12-21

**Authors:** Kazuyoshi Hosomichi, Takashi Shiina, Shingo Suzuki, Masayuki Tanaka, Sayoko Shimizu, Shigehisa Iwamoto, Hiromi Hara, Yutaka Yoshida, Jerzy K Kulski, Hidetoshi Inoko, Kei Hanzawa

**Affiliations:** 1 Department of Molecular Life Science, Division of Basic Medical Science and Molecular Medicine, Tokai University School of Medicine, 143 Shimokasuya, Isehara, Kanagawa 259-1143, Japan; 2 Laboratory of Animal Physiology, Faculty of Agriculture, Tokyo University of Agriculture, 1737 Funako, Atsugi, Kanagawa 243-0034, Japan; 3 Centre for Comparative Genomics, School of Information Technology, Murdoch University, Murdoch, WA 6150, Australia

## Abstract

**Background:**

The quail and chicken major histocompatibility complex (*Mhc*) genomic regions have a similar overall organization but differ markedly in that the quail has an expanded number of duplicated class I, class IIB, natural killer (NK)-receptor-like, lectin-like and BG genes. Therefore, the elucidation of genetic factors that contribute to the greater *Mhc *diversity in the quail would help to establish it as a model experimental animal in the investigation of avian *Mhc *associated diseases.

**Aims and approaches:**

The main aim here was to characterize the genetic and genomic features of the transcribed major quail *MhcIIB *(*CojaIIB*) region that is located between the *Tapasin *and *BRD2 *genes, and to compare our findings to the available information for the chicken *MhcIIB *(*BLB*). We used four approaches in the study of the quail *MhcIIB *region, (1) haplotype analyses with polymorphic loci, (2) cloning and sequencing of the RT-PCR *CojaIIB *products from individuals with different haplotypes, (3) genomic sequencing of the *CojaIIB *region from the individuals with the different haplotypes, and (4) phylogenetic and duplication analysis to explain the variability of the region between the quail and the chicken.

**Results:**

Our results show that the *Tapasin-BRD2 *segment of the quail *Mhc *is highly variable in length and in gene transcription intensity and content. Haplotypic sequences were found to vary in length between 4 to 11 kb. *Tapasin-BRD2 *segments contain one or two major transcribed *CojaIIBs *that were probably generated by segmental duplications involving c-type lectin-like genes and NK receptor-like genes, gene fusions between two *CojaIIBs *and transpositions between the major and minor *CojaIIB *segments. The relative evolutionary speed for generating the *MhcIIBs *genomic structures from the ancestral *BLB2 *was estimated to be two times faster in the quail than in the chicken after their separation from a common ancestor. Four types of genomic rearrangement elements (GRE), composed of simple tandem repeats (STR), were identified in the *MhcIIB *genomic segment located between the *Tapasin-BRD2 *genes. The GREs have many more STR numbers in the quail than in the chicken that displays strong linkage disequilibrium.

**Conclusion:**

This study suggests that the *Mhc *classIIB region has a flexible genomic structure generated by rearrangement elements and rapid SNP accumulation probably as a consequence of the quail adapting to environmental conditions and pathogens during its migratory history after its divergence from the chicken.

## Background

The genomic region of the major histocompatibility complex (*Mhc*) contains multi-gene family members involved in the immune response. The *Mhc *class I and class II genes encode glycoproteins that transport foreign peptides to the surface of cells for recognition by T cell receptors on lymphocytes, which in turn kill infected cells [1]. The *Mhc *class II molecules have highly polymorphic peptide binding regions (PBR) for the α1 and β1 domains encoded by the class IIA and class IIB genes, respectively, in various vertebrates including avian [2]. These polymorphisms may have been generated by gene conversion and positive selection, such as balancing selection and overdominant selection to adapt to life-environmental pathogens [3,4].

The most information currently available on the genomic organization of the *Mhc *in birds is for the chicken and quail. The chicken (*Gallus domesticus*) *Mhc *(*Gado*) region is divided into two major parts, *Gado-B *and *Gado-Y *[5]. Both of these regions are inherited independently of each other, although they are physically linked on micro-chromosome 16 (GGA16), [6-9]. From previous genomic studies, GGA16 is suggested to be an essential immunity chromosome, which is composed of genes involved with adaptive immunity (*BF/BL *segment in *Gado-B*), innate immunity (*Gado-Y*) and intrinsic immunity (*TRIM-like *and *BG *gene segments in the extended *Gado-B *region [[5,10-13], Shiina *et al*, Unpublished data].

The quail (*Coturnix japonica, Coja*) belongs to the same order (*Galliformes*) and family (*Phasianidae*) as the chicken. Whereas the quail is a migratory bird originating from Northern and Southern Asia, flying only short distances at a time and with a relatively short history of domestication, the chicken is a non-migratory bird originating from Southeastern Asia and with a thousand year history of domestication. Chicken – quail hybrids have been produced by artificial insemination of quails with chicken sperm [22]. Several immunological traits have been compared between various lines of quail which were selected for high and low IgY levels in the serum [23], and for high and low secondary antibody responses to Newcastle disease virus [24,25], influenza virus, sheep erythrocytes and *Salmonella pullorum *[26]. The quail is susceptible to Marek's disease virus as observed in the chicken. The chicken *Gado-B *complex has a significant influence in the genetics of disease resistance, such as Rous sarcoma and Marek's disease, but which genes within the complex are responsible and how they confer resistance or susceptibility is not known [16-18]. One important obstacle to elucidating the *Mhc *resistance genes in chicken is that the *BF*, *BL *and *BG *genes have co-evolved as haplotypes with a high linkage disequilibrium between the genes due to the compact structure of its "minimal essential" *Mhc *[19-21]. However, the immune response against the Marek's disease tumor-associated surface antigen (MATSA) in the quail differs significantly from that in the chicken [27]. Such immunological differences are likely to be due in part to the number and variation in *Mhc *gene loci and/or alleles, but the data concerning the *Coja *haplotypes and their association with disease is largely lacking. From our previous transcription and genomic studies, we found that the quail and chicken *Mhc *regions have a similar overall organization, but differ markedly in that the quail has an expanded number of duplicated genes with 7 *class I*, 10 *class IIB*, 4 *natural killer (NKr)-like receptor*, 6 *lectin-like receptor *and 8 *BG *genes [14,28,29]. To explain these findings, haplotypic genome comparison among quails and between the quail and the chicken is a matter of primary importance to elucidate the gene organization, molecular mechanism of polymorphism generation and disease analysis.

In order to better understand the genetic factors involved in the generation of *Mhc *diversity in quail, we identified and characterized six *CojaIIB *haplotypes by genotyping and polymorphism analysis using expressed *CojaIIB *sequences. We determined the genomic sequences within the *Tapasin-BRD2 *genomic segment of five different *Coja *haplotypes including the major transcribed *CojaIIBs*. We also compared the quail *Mhc *class II genomic structures and diversities of the chicken *B12 *and *B21 *haplotypic orthologs by phylogenetic analysis and duplication modeling. This study shows that the quail has much greater *Mhc *class IIB diversity and genomic structural flexibility than the chicken even though the two species are closely related in the evolutionary spectrum.

## Results and discussion

### *Coja *haplotype analysis with polymorphic loci

**Figure 1:**
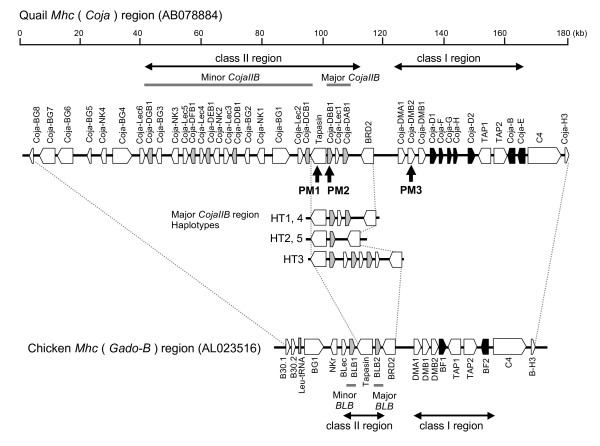
**Comparative gene map of quail and chicken *Mhc *region and locations of the genetic markers PM1 to PM3**. The map shows the comparison of the 180 kb *Coja *haplotype 1 sequence (AB078884: Shiina *et al *2004) and the 92 kb chicken *B12 *haplotype sequence (AL023516: Kaufman *et al *1999). Black and gray boxes indicate the *Mhc *class I and class IIB loci respectively. White boxes indicate other genes. The labeled vertical arrows indicate the locations of the newly designated genetic markers PM1 – PM3.

Three polymorphic markers PM1, PM2 and PM3, (Table 1) were used to genotype 48 randomly selected quails. We chose these three markers for the preliminary classification of *Coja *haplotypes because they are located in the *Tapasin*, *Coja-DBB1 *and *Coja-DMB2 *loci, respectively (Figure 1). The allele frequencies and heterozygosities for the polymorphic markers are presented in Table 2. All of the genotype frequencies were over 13% and the heterozygosity for each marker was over 0.65. The minimum number of haplotypes predicted from the maximum likelihood analysis of the 48 quails by the two different methods, a Bayesian statistical method and an Expectation-Maximization (EM) algorithm, was six (Table 3). Of the six haplotypes, the haplotype numbers (HT1 to HT5 had higher frequencies, ranging from of 14.6% to 28.1%, than HT6 (5.2%). Interestingly, HT2 and HT6 had the same "6–10" and "*02" allele combinations at the PM1 and PM2 loci respectively, but a different allele type at the PM3 locus (Table 3).

On the basis of finding the different allele type at the PM3 locus in HT6, recombination happened at least once in the region within the 24.4 kb segment between the quail genes *Coja-DBB1 *and *Coja-DMB2 *(PM2–PM3) and the newly created haplotype has spread in the population. Future analyses using fully pedigreed families should help to ascertain whether the intervening segments of DNA are the same in different individuals with the same three-marker genotype. Nevertheless, the variability in the quail genomic segment between the *Coja-DBB1 *and *Coja-DMB2 *genes corresponds to the chicken *BF*/*BL *region that displays strong linkage disequilibrium and genomic structural stability [19,20].

### Gene loci identification and haplotype reanalysis by RT-PCR, cDNA cloning and sequencing of transcribed *CojaIIB *loci

In order to identify and characterize the transcript sequences expressed by the gene loci of the different haplotypes, six quails with relatively high HT frequencies representing haplotypes HT1 to HT5 were selected for RT-PCR, cDNA cloning and sequencing analysis. The transcription intensity of the *CojaIIB *genes was estimated from the number of clones sequenced. A summary of the transcription intensity results for the five haplotypes is presented in Table 4. The Table shows the transcription intensity for HT1 to HT5 as a percent frequency of the clones for each individual quail (identified as numbers 302, 311, 312, 321, 322 and 323) with the percent frequency of clones per haplotype in parenthesis. The Table also shows the gene locus, the detected allele type, the PM2 allele, the nucleotide accession number in GenBank and the percent nucleotide similarity of the cDNA clones to the transcribed sequences expressed by the *CojaIIB *loci of HT1.

**Figure 2:**
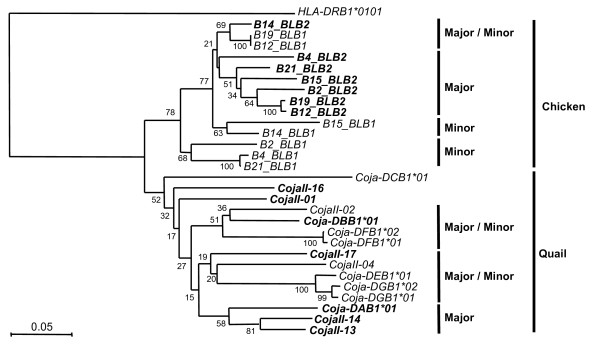
**Phylogenetic tree of the quail and chicken transcribed *MhcIIBs***. The dendrogram was constructed from the nucleotide sequences of the β1 extracellular domain regions (exon 2) (270 nucleotides in length) of *MhcIIB *genes for the 16 *CojaIIBs *shown in figure S1 the chicken haplotypic *BLB *sequences on *B2 B4 B12 B14 B15 B19 *and *B21 *(AJ248576 AJ248583 AJ248572 AJ248581 AJ248585 AJ248577 AJ248584 AJ248579 AJ248580 AJ248582 AJ248574 AJ248586 AJ248575 and AJ248573) and *HLA-DRB1 *(AF142457). The *HLA-DRB1 *sequence used as a species outlier roots the tree. Values near the branch-points of the tree indicate the bootstrap values. Bold letters indicate major transcribed *CojaIIBs *and *BLBs*.

In total, 15 kinds of *CojaIIB *cDNA sequences derived from the RT-PCR products of six quails were identified by sequence comparison (Table 4). Another 19 *CojaIIB *sequences were previously reported [14,25]. Altogether, these expressed sequences were identified to be expressed either by loci that were previously described alphabetically as *Coja-DAB1 to Coja-DGB1 *for HT1 or by as yet unnamed loci. The new loci identified in this study were therefore given names, such as *CojaII-01*, *CojaII-02*, *CojaII-13 *and *CojaII-16 *as listed in Table 4, using the naming formulae "*CojaII-XX*" as suggested by Shimizu *et al *[25] for the nomenclature of *CojaIIB *sequences. Each quail transcribed three to eight kinds of *CojaIIB *gene sequences (Table 4). Five to six of the cDNA sequences, which were observed in three quails (302, 312 and 321), perfectly matched with six of the seven *CojaIIB *loci of *Coja *haplotype 1 (HT1), that is *Coja-DAB1 *and *-DBB1 *in the major class II region, and *Coja-DCB1*, *-DEB1*, *-DFB1 *and *-DGB1 *in the minor class II region [14]. However, no sequences were detected in our study for the HT1 *Coja-DDB1 *locus. In addition, two other cDNA sequences were tentatively named Coja-DFB1*02 in HT5 and Coja-DGB1*02 in HT1 because they appear to be additional alleles at the *Coja-DFB1 *and *-DGB1 loci*, respectively, as determined from our phylogenetic analysis (Figs. 2, Table 4, Additional file 1). The sequences of *CojaII-01*, *-02 *and *-04 *were perfectly matched to the previously determined *CojaII-01HL*, *-02HL *and *-04H *haplotype sequences (Accession numbers; AB110476 to AB110478), respectively, which were derived from different inbred lines [25,26]. However, the *CojaII-13*, *-14*, *-16 *and *-17 *sequences did not have significantly high nucleotide similarities with the previously determined sequences (76.3 ~ 88.9%) of HT1 sequences and, therefore, were assigned as belonging to other unique haplotypes (Table 4).

On the basis of the correlation of the *Coja *haplotypes and distribution of the transcribed *CojaIIBs *for each of the quails, the fifteen *CojaIIB *sequences were classified to five distinct *Coja *haplotypes, namely, HT1, consisting of *Coja-DAB1*, *-DBB1*, *-DCB1*, *-DEB1*, *-DFB1 *and *-DGB1 *loci; HT2, *CojaII-13*; HT3, *CojaII-16 *and *-17*; HT4, *CojaII-01*, *-02 *and *-04*; and HT5, *Coja-DFB1 *and *CojaII-14 *(Table 4). From the cDNA cloned frequencies per *Coja *haplotype, *Coja-DAB1*, *-DBB1*, *CojaII-01*, *-13*, *-14*, *-16 *and *-17*, excluding *-DAB1 *in quail 321, were the major transcribed *CojaIIBs *with cloned frequencies of 34.8 ~ 100%, *CojaII-02 *and *-04 *were moderately transcribed *CojaIIB*s with cloned frequencies of 19.1 ~ 29.4%, and Coja-DCB1*01, -DEB1*02, -DFB1*01, -DFB1*02, -DGB1*01 and -DGB1*02 were minor transcribed *CojaIIB*s with cloned frequencies of 2.9 ~ 14.3% (Table 4). This result suggests that each *Coja *haplotype has at least one and up to seven transcribed *CojaIIB *loci with one or two of them representing the major locus.

### Genomic diversity of *Tapasin-BRD2 *segment

The *Tapasin-BRD2 *genomic segment contains the major *CojaIIB *region with the *Coja-DAB1*, *Coja-DBB1 *and the *Coja-Lec1 *genes flanked by the *Tapasin *and the *BRD2 *genes. This segment also has the *PM1 *and *PM2 *markers that we used for haplotyping. In order to study the genomic diversity of the *Tapasin-BRD2 *segments in different haplotypes by genomic sequencing, two cosmid libraries were constructed representing the haplotypes HT2, HT3, HT4 and HT5.

The average insert sizes of cosmid libraries constructed from the genomic DNA of quails 311 (HT 2/3) and 322 (HT 4/5) were estimated to average 37.5 kb and 44.6 kb, respectively, by 0.3% agarose gel electorophoresis analysis using 20 randomly selected cosmids (data not shown). As the quail has a genome size of 1.2 × 10^9 ^bp, similar to the chicken, the two cosmid libraries 311 and 322 were expected to cover 2.5 × 10^10 ^bp (20.9-fold) and 21.4 × 10^10 ^bp (17.8-fold), respectively [11]. Two *Tapasin *(clone number: 311CIIB-18 and 311CIIB-20) and two *BRD2 *positive cosmids (322CIIB-01 and 322CIIB-02) were isolated from HTs 2/3 and HTs 4/5 cosmid libraries, respectively. DNA typing revealed that the *CojaIIB *genes within the cosmids 311CIIB-18, 311CIIB-20, 322CIIB-01 and 322CIIB-02 corresponded to the haplotypes HT2, HT3, HT5 and HT5, respectively. Therefore, we selected the cosmids 311CIIB-18 (HT2), 311CIIB-20 (HT3) and 322CIIB-02 (HT5) for the genomic sequencing study of the *Tapasin-BRD2 *segment.

**Figure 3:**
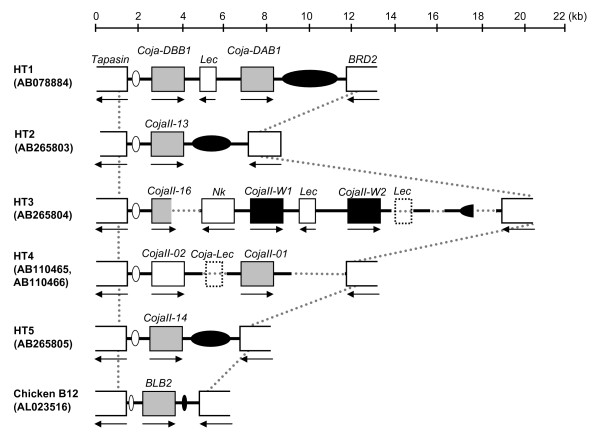
**Genomic structures of the major transcribed *MhcIIB *segments in the quail and chicken**. The map shows the comparison of the major transcribed *MhcIIB *segments on five *Coja *haplotypic sequences (HT1 HT2 HT3 HT4 and HT5) and the chicken *B12 *haplotype sequence. Gray striped and black boxes indicate the major transcribed *MhcIIB *moderately transcribed *MhcIIB *and non-transcribed *MhcIIB *respectively. White boxes indicate other genes. The numbers in parenthesis are the GenBank accession numbers. The white oval indicates TB1 and the black oval indicates the location of TB2-1 TB2-2 and TB3.

The genomic sequences derived from 311CIIB-18, 311CIIB-20 and 322CIIB-02 cosmids were determined by the shotgun method. Also a part of the *Tapasin-BRD2 *segment was sequenced from the long-ranged PCR products amplified from the genomic DNA of HT3 and HT4 homozygote quails [25,26]. The genomic structures of the major transcribed *MhcIIB *segments within the 5 quail haplotypes H1 to H5 and the chicken haplotype *B12 *derived from the genomic sequencing information and transcript alignments are shown in Fig. 3. The dot-matrix analyses between the 5 *Coja *haplotypes, chicken and quail, and two chicken haplotypes are shown in Additional file 2. After sequencing, the nucleotide lengths of the segment between the *Tapasin-BRD2 *genes were determined to be 5,984 bp in HT2, 16,462 bp in HT3, 5,222 bp in HT4 and 5,840 bp in HT5. The sequence of four segments on HT3 and HT4 was not determined fully however, for DNA structural reasons, such as the difficulties encountered with long repeat sequences and extremely high GC contents (Figs. 3, Additional file 2).

The *Tapasin-BRD2 *segment showed extremely complicated genome structures among the *Coja *haplotypes examined, similar to the *HLA-DR *region in the human *MHC *genomic region (Figs. 3, Additional file 1) [31]. The *Tapasin-BRD2 *segment of HT2 and HT5 contained only one major transcribed *CojaIIB *(*CojaII-13 *and *-14*, respectively) and their genomic structures and nucleotide length were similar to the chicken orthologous *BL *region (Figs. 3, Additional file 1). The *Tapasin-BRD2 *segment of HT1 contained two major transcribed *CojaIIBs *(*Coja-DAB1 *and *-DBB1*) (Fig. 3) [13]. Similarly, the *Tapasin-BRD2 *segment of the HT4 contained two *CojaIIB*s (*CojaII-01 *and *CojaII-02*) with major and moderately transcribed *CojaIIB *genes (Table 4). In HT3, the *Tapasin-BRD2 *segment contained three *CojaIIB*s, *CojaII-16*, *Coja-W1 *and *Coja-W2*. The *CojaII-16 *is a major transcribed *CojaIIB*, but although both *Coja-W1 *and *-W2 *have intact structures their mRNA was not obtained from the peripheral blood cells (Fig. 3). In all of the major transcribed *CojaIIBs*, the nucleotide length of intron 1 was variable whereas the other introns were relatively well conserved (Table 5). Moreover, one major transcribed *CojaIIB *(*CojaII-17*) that was observed in the HT3 (Table 4) was not identified in the *Tapasin-BRD2 *segment (Additional file 1). Consequently, the *CojaII-17 *is the only gene that either locates to a minor transcribed *CojaIIB *locus or to some other genomic region.

In summary, the *Tapasin-BRD*2 segment contains one or two major transcribed *CojaIIB*s that were identified in this study in much the same way as the chicken with its major transcribed *MhcIIB*, *BLB2 *(Figs. 1, 3). In contrast to *Xenopus *and mammals that also have *MhcIIB *genes located within the *Tapasin-BRD*2 segment, the bony fishes, such as medaka, fugu and rainbow trout, have *MhcI *genes within the *Tapasin-BRD*2 segment. Thus, the locations of the major transcribed *CojaIIBs *and *BLB2 *are well conserved in birds and more comparable with mammals and reptiles than with bony fish from the point of view of evolution [15,32-34].

### Molecular evolutionary analysis of the chicken and quail *MhcIIB *genomic region

In a comparison of the *CojaIIB *gene organization of different haplotypes, the *CojaIIB *genomic units in the *Tapasin-BRD2 *segment of the HT1 [*DBB1 *– *Lec *– *DAB1*] and HT4 [*CojaII-02 *– *Lec *– *CojaII-01*] were noted to resemble the minor *CojaIIB *genomic *DFB1/DEB1 *unit [*DFB1 *– *Lec4 *– *DEB1*], whereas those of the HT3 [*CojaII-W2 *– *Lec *– *CojaII-W1 *– *Nkr *– *CojaII-16*] resemble the *DFB1/DEB1/DDB1 *unit [*DFB1 *– *Lec4 *– *DEB1 *– *NK2 *– (*Lec3*) – *DDB1*], although they are in the opposite direction to each other (Fig. 3). Thus, two kinds of successive *trans-*segmental duplications involving non-*Mhc *genes appear to have produced the major *CojaIIB *segments independently. However, significant nucleotide homologies between the duplicated units were not observed (data not shown). Since comparable segmental duplication variability has not been observed in the *Gado-B *region, the *trans*-segmental duplications in quail are likely to have occurred after speciation of the quail and chicken from their common ancestor. In the case of human *MHC*, the traits of segmental duplications and transpositions were observed in the *HLA *class I region [35], and especially the generation of the *HLA-B *and *-C *segment that is explained by the events involving the *MHCI*, *MIC*, *HCGII*, *HCGIV*, *HCGIX*, *HCP5*, *3.8-1 *unit in the evolutionary process [36].

To clarify the genetic relationships among the *CojaIIB *sequences and between the quail and the chicken *MhcIIB *sequences, a phylogenetic tree of exon 2 was constructed (Fig. 2). This tree suggests that the *CojaIIB *gene sequences are more closely related to each other in the quail than to the chicken *MhcIIB *(*BLBs*), and that the *CojaIIB *genes have been duplicated after speciation of quail and chicken. The average genetic distance of the *CojaIIB *sequences (0.194+0.018) after speciation is twice as long as that of the *BLB *sequences (0.107+0.016), meaning that the *CojaIIB *genes have a relatively faster evolutionary speed than the *BLBs *(Fig. 2). On the other hand, chicken haplotype sequences are more closely related. From the genome sequencing of the Red Jungle Fowl *Mhc *region, the *BLB *nucleotide sequences were perfectly matched with domestic chicken B21*BLB1 and B21*BLB2 sequences [Shiina *et al*, Unpublished data]. Therefore, the *BLB *genes appear to have been generated from a recent common ancestor of both the egg-laying domestic chicken and broilers.

**Figure 4:**
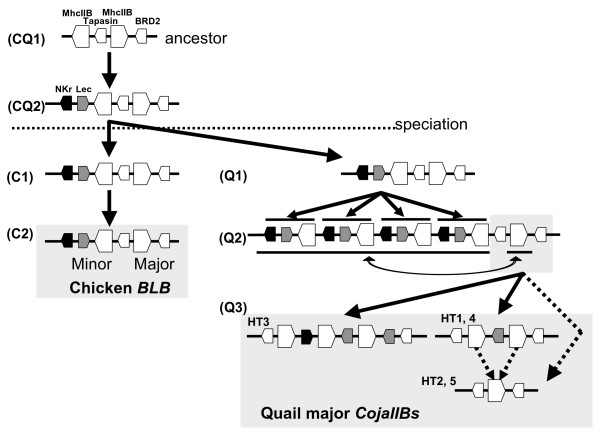
**Gene duplication models inferred from the reconstructed phylogenetic trees**. The labeled arrows indicate the speciation and gene duplication events for the chicken (C) and quail (Q) lineages. Dotted arrows indicate the evolutionary process for the origin of HT2 and HT5. Gray backgrounds indicate quail and chicken *MHCIIB *structures at the present-day.

In the phylogenetic tree, the quail lineage was divided into three main clusters, one major and two major/minor intermingled clusters, with the Coja-DCB1*01, *CojaII-01 *and *CojaII-16 *out-grouped from the three main clusters. In comparison, the chicken lineage was divided into four main clusters, namely one major, two minors and one major/minor intermingled cluster (Fig. 2). In the case of the major transcribed *CojaIIBs*, these were observed in all clusters. In addition, the traits for two kinds of segmental duplications were observed in the *Tapasin-BRD2 *segment as previously mentioned, suggesting that the major transcribed *CojaIIB*s were generated by independent duplications and/or transpositions between the major and minor *CojaIIB *segments at a relatively high evolutionary speed after the separation of the quail and chicken from their common ancestor (Fig. 4). In contrast, the *BLB2 *sequences of extant chickens appear to have been generated from an ancestral *BLB2 *gene with very little structural reorganization or change (Fig. 4).

### Genomic rearrangement elements in the major *CojaIIB *segment involved in the generation of *Mhc *diversity

Four types of tandem repeat sequences were identified that possibly drive genomic rearrangement events within the *Tapasin-BRD2 *segment of HT1, HT2, HT3 and HT5 (Table 6). These simple candidate rearrangement elements are TB1, a known T-cell factor motif; TB3 and TB3, STRs found in the mouse *Mhc *class II region; and TB4, a recombination motif [37-39]. From the comparison of the repeat numbers of the solitary repeat unit between quail and chicken, the *Coja *haplotypes were found to contain numerous repeat numbers for TB1 to TB4, whereas the chicken *B12 *and *B21 *haplotypes were found to have none or only a few repeat numbers (Table 6). The TB repeats were not identified in the minor transcribed *CojaIIB *segment of the HT1 genome contigs [14]. Therefore, the TB repeats appear to be rearrangement elements within the genome that provide strong driving forces for the generation of new major transcribed *CojaIIBs *via duplications and/or transpositions.

### Genomic diversity within the major *CojaIIB *region and disease studies

*Mhc *diversity in vertebrates is often attributed to the high SNP content in this genomic region. The generation of nucleotide polymorphism of *Mhc *genes is usually explained by positive selection, such as balancing selection and overdominant selection acting on the *Mhc *genes, which is necessary to maintain polymorphisms for survival against infections [3,4]. These selected pressures also lead to a hitch-hiking effect that results in the accumulation of many additional SNPs around the *Mhc *gene [30]. Namely, the DNA segments affected by hitch-hiking have arisen due to the accumulated effect of overdominant selection and balancing selection. In the *HLA *region, *HLA-A*, *-B*, *-DR/DQ *and *-DP *are thought to have been affected by hitch-hiking and associated with several diseases, such as IDDM, rheumatoid arthritis and psoriasis vulgaris [30,40]. The hitch-hiking effect was also observed in *Gado-B *region [Shiina *et al*, Unpublished data]. Because the *CojaIIBs *have developed at a faster evolutionary speed than the *BLBs *(Fig. 2), the *CojaIIB *region is likely to be also effected by hitch-hiking. If a harmful variation is generated around the *Mhc *gene via the hitch-hiking effect, then it is likely to be selected against by genetic recombination.

A serious problem for disease mapping in the *Gado-B *region is that genetic recombination within the *BF, BL *and *BG *loci is rarely observed under experimental conditions, Therefore, *Gado-B *haplotypes encompassing alleles in all three loci are the units of inheritance most often considered in relating the *Gado-B *complex to immunity and disease responses [5]. On the other hand, the quail appears to be relatively more resistant than the chicken to many viral diseases [41]. In addition, the quail is thought to play an important role in the evolution of influenza viruses by acting as an intermediate host in which avian influenza viruses can be amplified and transmitted to other animal species [42]. Since the *Coja *region has the duplication and divergence of *CojaIIBs *(Figs. 2, 3, Additional file 1), the *Coja *region may be a superior system for the selection of beneficial variations enabling more antigen presentation ability than the *Gado-B *region. Therefore, to identify the disease genetic factors associated with the *Gado-B *region, comparative genomic and disease mapping analysis of the *Coja *region is also important.

## Conclusion

In conclusion, we characterized the genetic and genomic features of the *CojaIIB *region and obtained the following three main findings. Firstly, one to six transcribed *CojaIIB *loci were identified in each *Coja *haplotype, of which one or two of them was the major coding locus. The major *CojaIIB *genes, except for *CojaII-17 *of HT3, were located within the *Tapasin-BRD2 *segment. In contrast to the quail, the chicken has one major and one minor *MhcIIB*. Secondly, phylogenetic and evolutionary analyses suggest that the major transcribed *CojaIIB*s were organized by independent *trans*-segmental duplications involving non-*Mhc *genes and/or transpositions between major and minor *CojaIIB*s. Consequently, the *CojaIIBs *have a relatively faster evolutionary speed than the *BLBs*. In contrast to the quail, the chicken *BLB2 *alleles have been generated from the ancestral *BLB2 *probably since the separation of chickens and quails from their common ancestor. Thirdly, four types of genome rearrangement elements (remodeling repeats) composed of tandem repeats were identified within the 4 ~ 11 kb haplotypic *Tapasin-BRD2 *segment, and the quail has far more repeat numbers for rearrangement events than the chicken that displays strong linkage disequilibrium [19-21]. Taken together, these three main findings support the view that the genomic diversity of the *CojaIIB *region has been generated by duplications and gene transpositions via the candidate rearrangement elements along with a fast evolutionary speed in adapting to environmental conditions and pathogens during the migratory history of the quail after its divergence from the chicken. It is evident from our study that the quail *MhcIIB *region has a much more flexible genomic structure than the chicken for generating greater *Mhc *diversity.

## Methods

### Quail

Blood samples were collected from 48 randomly selected quails maintained at the Tokyo University of Agriculture [23,43]. The blood collection and animal studies were conducted in accordance with the Guidelines for Animal Experiments at the Tokyo University of Agriculture.

### Genotyping and haplotype analyses

Three polymorphic markers, PM1 to PM3, were identified in the *Mhc *classIIB genomic region of the quail (Figure 1) and were used as markers in this study for an initial haplotype analysis [14] (Tables 1, 2) either by PCR and sequence based typing or by PCR and RFLP analysis. PM1 is a mini-satellite marker composed of two different types of repetitive sequences and located in the *Tapasin *gene; PM2 is *Coja-DBB1 *locus genotyping marker; and PM3 is composed of two SNP markers located within the *Coja-DMB2 *gene (Table 1). Five different PM1 alleles were first identified by sequencing and then correlated with the length of their PCR products. The PCR product sizes of the PM1 alleles were then used to identify and define the alleles in the individual samples without the need for further sequencing. The PM1 alleles were designated as "6–10", "8–12", "9–12", "10–14" and "18–14" based on the length of the repeat units. For example, the "6–10" allele corresponds to the sequence TTCCTATGGGGGCTGTAGGGTGGATGGGACTGGGTGGTA^6 ^– ATGAT^10^. Five PM2 alleles (Table 2) amplified by PCR were detected by a sequence based typing method of the PCR products and designated as Coja-DBB1*01 (alias "*01"), "*02", "*03", "*04" and "*05". The three PM3 alleles designated as alleles "G-C", "G-G" and "A-C" were detected by a PCR-RFLP method using *Dra*III and *Hin*fI (Table 1) to identify the SNPs "G/A" and "G/C" at the two SNP sites that are apart by 194 bp. Haplotype prediction was performed by PHASE and Arlequin programs [44,45]. Maximum-likelihood haplotype frequencies were predicted by a Bayesian statistical method of the PHASE program and an EM algorithm of the Arlequin program.

### RT-PCR, cloning cDNA and sequence determination

Total RNA was isolated from the peripheral blood of six quails identified by the numbers302, 311, 312, 321, 322 and 323 and having the haplotypes (HTs) 1/1, 2/3, 1/3, 1/4, 4/5, and 2/4 respectively. The TRIzol reagent was used to isolate the total RNA as described by the manufacturer (Invitrogen, Groningen, Netherlands). Total RNA (0.2 ug) was synthesized to cDNA by using First Strand cDNA Synthesis Kit (Rever Tra Ace-α-) and the oligo (dT) primer method (TOYOBO, Osaka, Japan). The *CojaIIB *specific primers were designed to amplify the exons 1 – 3 (amplified size: from 411 bp to 441 bp) and to detect polymorphisms on hyper variable β1 domain (exon 2) by RT-PCR amplification with the sense primer (C2BNO2: 5'-GAGTGCCACTACCTGAACGGCACCGAGG-3') and the anti-sense primer (C2BNO5: 5'-GCGCCAGGAAGACGAGCCCCAGCAC-3'). These primer sequences were perfectly matched with all *CojaIIB *genes on the *Coja *haplotype 1 to 5 in this study, providing confidence that the primers will amplify all the known *CojaIIB *genes with the same efficiency. cDNA (10 ng) was amplified by PCR with 2.5 units of GeneTaq NT polymerase (Nippon Gene, Toyama, Japan), using the thermal cycler GeneAmp PCR system 9700. The reaction mixture (10 ul) was subjected to 30 cycles of 30 sec at 96°C, 30 sec at 65°C, and 30 sec at 72°C. The RT-PCR products were cloned into the pGEM-T Easy vector with the TA cloning kit according to the protocol provided by the manufacturer (Promega, Madison, WI, USA) and sequenced using the ABI3100 genetic analyzer (Applied Biosystems, Foster City, CA, USA) in accordance with the protocol of BigDye terminator method. To avoid PCR and sequencing artifacts generated by polymerase errors, 96 clones per individual were sequenced.

### Construction and screening of cosmid libraries

Genomic DNAs for constructing the cosmid libraries were isolated from the red blood cells of two *Coja *haplotype heterozygote quails (311 and 322), having HTs 2/3 and 4/5, by the Saponin-NaCl method [43]. The libraries were constructed by the SuperCos 1/Gigapack XL cloning kit according to the manufacturer's protocol (Stratagene, La Jolla, CA, USA). Approximately 5 × 10^5 ^independent colonies derived from these libraries were screened by using PCR products as hybridization probes obtained from the 527 ~ 586 bp *CojaIIB *fragment generated by the sense primer C2BNO2 and the anti-sense primer C2BNO5 already described above in the RT-PCR section; the 276 bp *Tapasin *fragment generated by sense primer Tapasin/e5-1 (5'-CCCAAAGAACCTGGTGGTGA-3') and anti-sense primer Tapasin/e5-4 (5'-AATGACCGTGGGTGACAA-3') and the 134 bp *BRD2 *fragment produced by the sense primer RING3/e4-1 (5'-GCAGCGGAGTGCAGAGACTT-3') and anti-sense primer RING3/e4-3 (5'-CGAGTGCCAGCTGTCTCCTC-3').

### Genomic sequencing strategy

The DNA of *CojaIIB*, *Tapasin *and *BRD2 *positive cosmids was sequenced by the shotgun method [46]. Individual sequences were minimally edited to remove vector sequences, and assembled into a contig using the Sequencher software (Gene Codes Co., Ann Arbor, MI, USA). Remaining gaps or ambiguous nucleotides were determined by the direct sequencing of the PCR products obtained with appropriate PCR primers or by nucleotide sequence determination of additional shotgun clones.

### Sequence analyses

Nucleotide similarities among the sequences were calculated by using the GENETYX-MAC software ver 11.0 (Software Development Co. Ltd., Tokyo, Japan). Dot-matrix analysis was performed by using HARRPLOT Ver. 2.0 as part of the GENETYX package. Multiple sequence alignments were created using the ClustalW Sequence Alignment program at DDBJ [47]. The phylogenetic tree was constructed by neighbor-joining method of the Molecular Evolution Genetics Analysis (MEGA3.1) [48].

## Authors' contributions

KHos, TS, HI and KHan participated in the design of this study. KHos and TS constructed the quail cosmid libraries. KHos, TS, SSuz, MT, SShi, SI, HH, YY, HI and KHan participated in sequencing and contig assembly of the cosmid clones and data analysis. KHos, TS, HI, JKK and KHan prepared this manuscript. All authors have read and approved the final manuscript.

## Supplementary Material

Additional file 1 Alignment of transcribed *CojaIIBs *for phylogenetic analysis. the nucleotide sequences of the β1 extracellular domain regions (exon 2) (270 nucleotides in length) of *MhcIIB *genes for the 16 *CojaIIBs *the chicken haplotypic *BLB *sequences on *B2 B4 B12 B14 B15 B19 *and *B21*. Click here for file

Additional file 2 Dot-matrix analysis among five *Coja *haplotypes (A ~ J) between quail and chicken (K) and between chicken *B12 *and *B21 *(L). Dot plot comparisons shows HT1 vs HT2 (A) HT1 vs HT3 (B) HT1 vs HT4 (C) HT1 vs HT5 (D) HT2 vs HT3 (E) HT2 vs HT4 (F) HT2 vs HT5 (G) HT3 vs HT4 (H) HT3 vs HT5(I) HT4 vs HT5 (J) HT2 vs chicken *B12 *(K) and chicken *B12 *vs *B21 *(L). Numbers and blue circles in the images show the location of genome candidate remodeling (rearrangement) factors with the numbers 1 to 3 representing TB1 – TB3 respectively as outlined in Table 6. Gray background shows the gap within the segments that was determined by sequencing. Click here for file

## Figures and Tables

**Table 1:** Basic features of the three polymorphic markers on *Coja *region

	*Coja *haplotype 1 (Acc. Num. AB078884)						
					
marker name	Nuc. position**	Gene position	length (bp)	range (bp)	polymorphisms	primer name	primer sequence (5' to 3')
**PM1**	89043 – 89887	*Tapasin *(ex.4 – int.4)	845	718–1095	(ATGAT)**n** (TTCCTATGGGGGCTGTAGGGTGGATGGGACTGGGTGGTA)**n**	TAPBPL-F	CAGGTCCTGCTGGCCTATGA
						TAPBPL-R	TGGTGTGATGCCAACCCAT
**PM2**	92768 – 93787	*Coja-DBB1 *(Promoter – ex.3)	1002	989–1002	126 SNPs, 13 indels	CojaDBBpromoterF6	CCCTGGGGACACCATTTGTCAT
						C2BNO5	GCGCCAGGAAGACGAGCCCCAGCAC
PM3*	118204 – 118536	*DMB2 *(int.1 – int.2)	333	333	G/A, G/C	DMB2-F	GGAGTGCATCCCCATTGCT
						DMB2-R	GCTCACTCTTGCGCAGTGC

* PM3 contains two SNP sites.

** positions on *Coja *haplotype 1 genomic sequence (accession number; AB078884).

**Table 2:** Genotype frequency and heterozygosity for three markers

marker	Allele	length (bp)	Nt. Acc, No.	Frequency (%)	Heterozygosity
PM1	9–12	845	AB282647	28.1	0.794
	6–10	718	AB282648	22.9	
	8–12	806	AB282649	13.5	
	10–14	900	AB282650	20.8	
	18–14	1095	AB282651	14.6	
PM2	*01	1002	AB282652	28.1	0.794
	*02	1001	AB282653	22.9	
	*03	989	AB282654	13.5	
	*04	1001	AB282655	20.8	
	*05	989	AB282656	14.6	
PM3	G-C	333	AB186377	28.1	0.650
	G-G	333	AB186378	45.8	
	A-C	333	AB186379	26.0	

**Table 3:** Summary of *Coja *haplotypes (HT1–6) and their frequencies

HT num.	PM1	PM2	PM3	Obs. Num.	Freq. (%)
1	9–12	*01	G-C	27	**28.1**
2	6–10	*02	G-G	17	**17.7**
3	8–12	*03	G-G	13	**13.5**
4	10–14	*04	A-C	20	**20.8**
5	18–14	*05	G-G	14	**14.6**
6	6–10	*02	A-C	5	5.2

Bold letter shows *Coja *haplotype frequency with over 10%.

**Table 4:** Percent frequency of cloned cDNA, distribution and classification of the transcribed *CojaIIB *allele as five distinct *Coja *haplotypes

HT number	Locus	Allele	PM2 alleles	Nt. Acc, No.	Nucleotide similarity to HT1 (%)	% frequency of cDNA clones per individual quail (% frequency of cDNA clones per haplotype)					
						
						quail individual number					
						
						302*	311	312	321	322	323
	
HT1	*Coja-DAB1*	Coja-DAB1*01		AB181861	100	46.9** (**46.9**)***	-	17.1 (**37.1**)	5.9 (19.0)	-	-
	*Coja-DBB1*	Coja-DBB1*01	Coja-DBB1*01	AB181862	100	35.9 (**35.9**)	-	22.4 (**48.6**)	16.2 (**52.4**)	-	-
	*Coja-DCB1*	Coja-DCB1*01		AB181863	100	6.3 (6.3)	-	1.3 (2.9)	2.9 (9.5)	-	-
	*Coja-DDB1*	-		-	-	-	-	-	-	-	-
	*Coja-DEB1*	Coja-DEB1*01		AB181865	100	4.7 (4.7)	-	2.6 (5.6)	-	-	-
	*Coja-DFB1*	Coja-DFB1*01		AB181866	100	-	-	1.3 (2.9)	1.5 (4.8)	-	-
	*Coja-DGB1*	Coja-DGB1*01		AB181868	100.0	6.3 (6.3)	-	-	4.4 (14.3)	-	-
	*Coja-DGB1*	Coja-DGB1*02		AB181869	98.9 (DGB1)	-	-	1.3 (2.9)	-	-	-
HT2	*CojaII-13*	CojaII-13*01	Coja-DBB1*02	AB181874	76.3 (DCB1) ~ 87.4 (DAB1)	-	62.9 (**100**)	53.9 (**100**)	-	-	67.2 (**100**)
HT3	*CojaII-16*	CojaII-16*01	Coja-DBB1*03	AB264281	78.9 (DCB1) ~ 85.2 (DBB1)	-	12.9(**34.8**)	-	-	-	-
	*CojaII-17*	CojaII-17*01		AB264282	77.5 (DCB1) ~ 83.3 (DGB1)	-	24.2 (**65.2**)	-	-	-	-
HT4	*CojaII-02*	CojaII-02*01	Coja-DBB1*04	AB181871	78.1 (DCB1) ~ 88.9 (DBB1)	-	-	-	13.2 (19.1)	14.5 (29.4)	-
	*CojaII-01*	CojaII-01*01		AB181870	78.9 (DCB1) ~ 87.8 (DBB1)	-	-	-	36.8 (**53.2**)	21.7 (**44.1**)	-
	*CojaII-04*	CojaII-04*01		AB181872	77.8 (DCB1) ~ 84.4 (DBB1)	-	-	-	19.1 (27.7)	13.0 (26.5)	-
HT5	*CojaII-14*	CojaII-14*01	Coja-DBB1*05	AB181876	77.4 (DCB1) ~ 87.4 (DAB1)	-	-	-	-	47.8 (**94.3**)	28.1 (**85.7**)
	*Coja-DFB1*	Coja-DFB1*02		AB181867	99.3 (DFB1)	-	-	-	-	2.9 (5.7)	4.7 (14.3)
						
	cDNA sub-clone					64	62	76	68	69	64
	Presumptive HT					1/1	2/3	1/2	1/4	4/5	2/5

Bold letter indicates sub-clone frequency over 30%. (-) means not transcribed. Coja-DFB1*02 was categorized to "HT5" because this allele sequence was observed in only HT5.

**Table 5:** Comparison of nucleotide length of intron and exons among *CojaIIB *in the *Tapasin-BRD2 *segment

Coja HT	HT1		HT2	HT3	HT4		HT5
					
*CojaIIB *PM2 alleles	*Coja-DAB1*	Coja-DBB1 Coja-DBB1*01	CojaII-13 Coja-DBB1*02	CojaII-16 Coja-DBB1*03	*CojaII-01*	CojaII-02 Coja-DBB1*04	CojaII-14 Coja-DBB1*05
	
exon1	91	91	91	91	91	91	91
intron1	30	**88**	**88**	76	146	**88**	76
exon2	270	270	270	270	270	270	270
intron2	86	86	86	86	86	86	86
exon3	282	282	282	282	282	282	282
intron3	95	95	95	95	95	95	95
exon4	111	111	111	111	111	111	111
intron4	76	76	76	76	76	77	76
exon5	24	24	24	24	24	24	24
intron5	**73**	86	**73**	**73**	**73**	**73**	**73**
exon6	14	14	14	14	14	14	14
total	1152	1223	1210	**1198**	1268	1211	**1198**

**Table 6:** Candidate rearrangement elements (TB1–4) identified within the *Tapasin-BRD2 *segment

Name	Features and sequence of repeat unit	Quail					Chicken	
			
		HT1	HT2	HT3	HT4	HT5	*B12*	*B21*
	TCF-I [37]							
TB1	MAMAG^n^	13*	13	26	27	23	3	3
	STRs in H-2 class II region [38]							
TB2	CAGA^n^	86	54	>14	Un	56	5	5
TB3	CAGG^n^	120	75	>21	Un	99	6	6
	Recombination hotspot motif [39]							
TB4	GGGCAGGARG^n^	9	4	>1	Un	5	1	1
	Total	228	146	>62	>27	183	15	15

* number of repeat units.
