# Supplementary material for: The major histocompatibility complex (Mhc) class IIB region has greater genomic structural flexibility and diversity in the quail than the chicken
Source: BMC Genomics. 2006 Dec 21;7:322. doi: 10.1186/1471-2164-7-322 (PMC1769493; doi:10.1186/1471-2164-7-322)
Supplement: Additional file 1 — Alignment of transcribed CojaIIBs for phylogenetic analysis. the nucleotide sequences of the β1 extracellular domain regions (exon 2) (270 nucleotides in length) of MhcIIB genes for the 16 CojaIIBs the chicken haplotypic BLB sequences on B2 B4 B12 B14 B15 B19 and B21. [file 1471-2164-7-322-S1.pdf]

Additional file 1. Alignment of transcribed *CojaIIBs* for phylogenetic analysis

```
Coja-DAB1*01    1    CGTTCTTCCTCTTCAGTATGCTGTCTGAGTGCCAGTTCTTCAACGGCACCGAGCGGGTAAGGTTGACGCAGGGGTACATCTACAACCGGCAGCAGTACAC    100
Coja-DBB1*01    1    ...A.....AG..TTA.GCTAGA...............C......A............G..A..TGT.GT..............................    100
Coja-DCB1*01    1    .........AA.GGCTGGGTAGAG.G..G.....T...C.G.........C.......G..A.ATGTAG.TA..C.......T.............TA.T    100
Coja-DEB1*01    1    ....G....AG.GGTC.T.TA.AG.G........C...C...........C.......G..A...GT.GT..............................    100
Coja-DFB1*01    1    ....T...TAT..GTC.CACAGA..GC.G.......A.C...................G....AT........A..........................    100
Coja-DFB1*02    1    ....T...TAT..GTC.CACAGA..GC.........A.C...................G....AT......A.A..........................    100
Coja-DGB1*01    1    ....G....AG.GGTC.T.TA.AG.G..G.....C...C...................G..A..TGT.G.TAC..........................T    100
Coja-DGB1*02    1    ....G....AG.GGTC.T.TA.AG.G..G.....C...C...................G..A..TGT.G.TAC......................AT...    100
CojaII-01       1    ...A.....AGCATTC.T.TA.A..G........C...C.G.........C.......G......GT.G.CC....T.......................    100
CojaII-02       1    ........AAG...TC.CATAGA.GG.......GC..TC.G.................G..A...GT.GC..............................    100
CojaII-04       1    .........A.CA....TC..CAA..........C...C.G.........C.......G..A..AGT.G.CA.....................C.CT...    100
CojaII-13       1    ...A.....A.CA....GC..AAA..........C...C.G.........C.......G.....................................T.G.    100
CojaII-14       1    .........AACATTC.T..A.TGGG.....T..C...C.G.........C.......G.....................................T.G.    100
CojaII-16       1    .........AACAT.C.T.TA.TGGG.....T..C...C.G.................G..A..TGT.GT.A............................    100
CojaII-17       1    .........A.CA....GC..AAAG...T...T.C...C...........C.......G..AC.TCT.G.TA....T...................T...    100
                     ***..***..  .   ...    ..  .  ...*.**....*...*.****.****.*******.**..........  ...*.*****.**********.*.....

Coja-DAB1*01    101  ACACTTCGACAGCGACGTGGGGAAATTCGTGGCCGATTCAACGCTGGGAGAGCGTCCGACTGAGTACTTAAACAGCCAAACCGAGTTTCTGGAGTACTTA    200
Coja-DBB1*01    101  G..........................T.........A..C............T..A.G.C...C...AC................A.........TAC.    200
Coja-DCB1*01    101  G.........................AT............C.......G....TG.AAG.....C....C......G.C.......A..........AA.    200
Coja-DEB1*01    101  G..........................T.........A..C..A........A..TG.G.....G....C......G.T.......A.AC......T.C.    200
Coja-DFB1*01    101  G...A...........T..........T.........A..C...........TT..A.G.....C...AC................A.......GT.AA.    200
Coja-DFB1*02    101  G...A...........T..........T.........A..C...........TT..A.G.....C...AC................A.......GT.AA.    200
Coja-DGB1*01    101  G..........................T.........A..C..A........A..TG.G.....G....C......G.T.......A.AC......T.C.    200
Coja-DGB1*02    101  G..........................T.........A..C..A........A..TG.G.....G....C......G.T.......A.AC......T.C.    200
CojaII-01       101  G.........................AT.........A..C...............A.G.........GG...............A........G.TGC.    200
CojaII-02       101  G..........................T.........A..............TT..A.G.....C..CAC..............................    200
CojaII-04       101  G..........................T.........A..C..A........A..TG.G..A..G....C......A.C.......A.......CGTGC.    200
CojaII-13       101  G..........................T........................A..TG.G.....G..G.C..............................    200
CojaII-14       101  G..........................T........................A..TG.G.....G..G.C..............................    200
CojaII-16       101  G...............T..........T.........A..C.................G.........GG..T...G.C......GAAA.......TAG.    200
CojaII-17       101  G..........................T.........A..C..A........A..TG.G.....G...GG........G.........A.....GT.AA.    200
                     .****.**********.*********..*********.**.**.****.***   ...   ..*..** **...**.***.*.******.....****...   *

Coja-DAB1*01    201  CGGGGTGTAGTGGACACGTGCTGCCGGCACAACTACGAGATTCTGGAGTCCATCACGGTGCAGAGGAGCG    270
Coja-DBB1*01    201  .......C...........A.................G.G.GT.T.........................    270
Coja-DCB1*01    201  .....ACAG.......G..AT.......TT.......G.GCG..T...C..G.A...C............    270
Coja-DEB1*01    201  ......TC...........T.......GT.GC..........G........T..................    270
Coja-DFB1*01    201  ....CA.A...........T.................G.G.GT.C.........................    270
Coja-DFB1*02    201  ....CA.A...........T.................G.G.GT.C.........................    270
Coja-DGB1*01    201  ......T............T.......GT.GC..........GG.......T..................    270
Coja-DGB1*02    201  ......T............T.......GT.GC..........GG.......T..................    270
CojaII-01       201  ....A..C...........A.......T.........G.G.GGAT...C..T..................    270
CojaII-02       201  .......C..........GT..................................................    270
CojaII-04       201  ...A...C.......TG..A.......T.........G.G.GT.C.........................    270
CojaII-13       201  .......C...........C.......T.........G.G..GGA......T..................    270
CojaII-14       201  .......C...........T............................C..T..................    270
CojaII-16       201  A..AA..AG..........T.................G.G.GT.C......T..................    270
CojaII-17       201  ....CA.A..........GT...............................T..................    270
                     .**....  .******..*...******.....*****.*...  .  ***.**.*.****.***********
```
