# Supplementary material for: The major histocompatibility complex (Mhc) class IIB region has greater genomic structural flexibility and diversity in the quail than the chicken
Source: BMC Genomics. 2006 Dec 21;7:322. doi: 10.1186/1471-2164-7-322 (PMC1769493; doi:10.1186/1471-2164-7-322)

Additional file 2. Dot-matrix analysis among five *Coja* haplotypes (A ~ J), between quail and chicken (K) and between chicken *B12* and *B21* (L).

(A) HT1 vs HT2

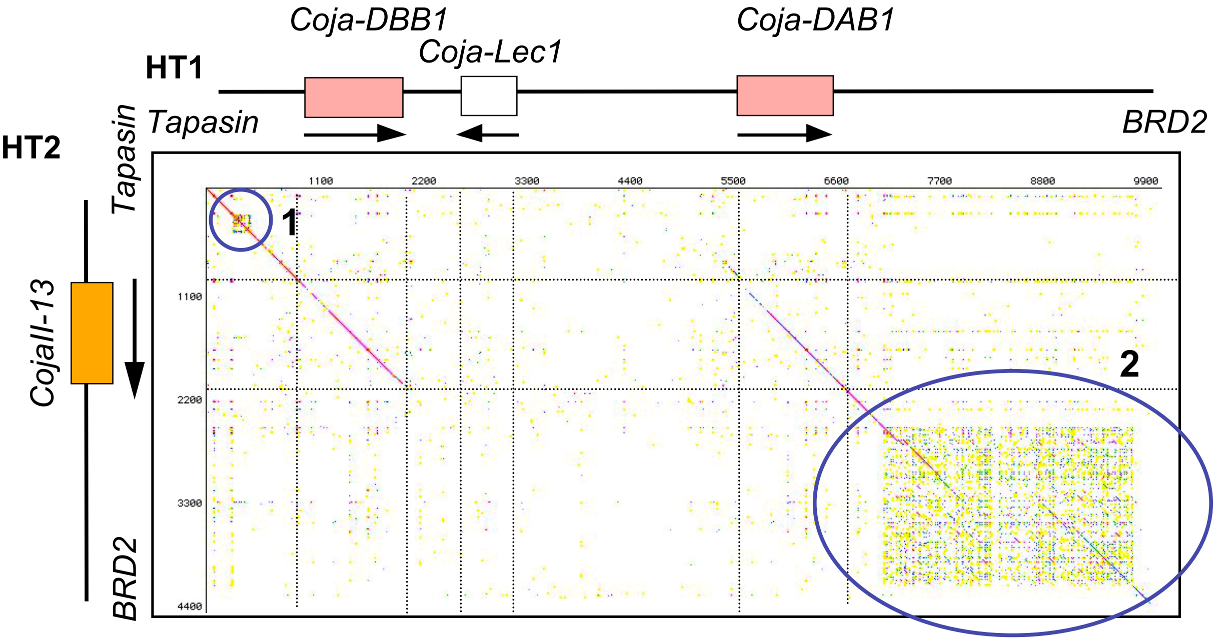

(B) HT1 vs HT3

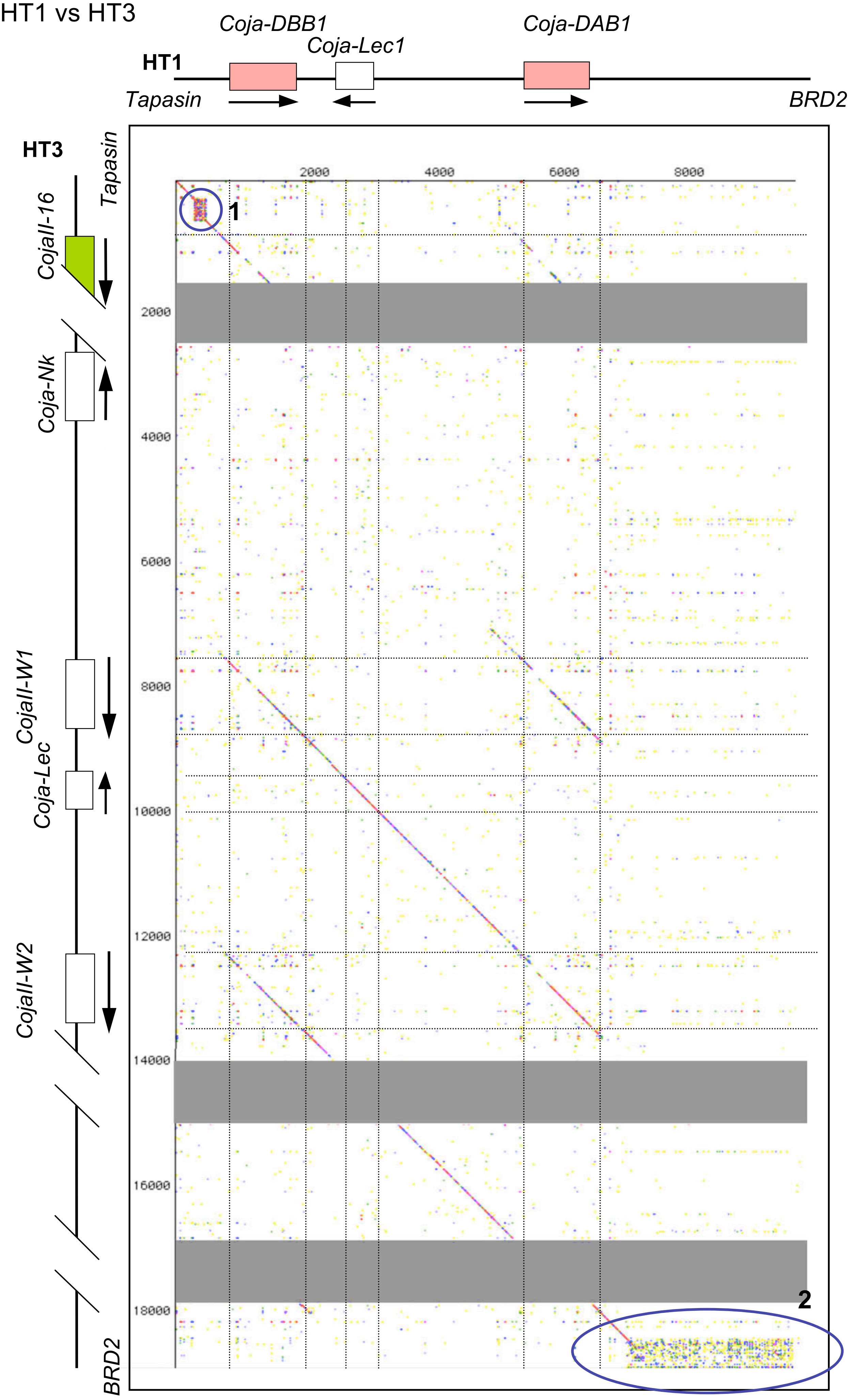
Coja-DBB1
Coja-Lec1
Coja-DAB1
HT1
Tapasin
BRD2
HT3
Tapasin
CojaII-16
Coja-Nk
CojaII-W1
Coja-Lec
CojaII-W2
BRD2
2000
4000
6000
8000
2000
4000
6000
8000
10000
12000
14000
16000
18000
1
2

(C) HT1 vs HT4

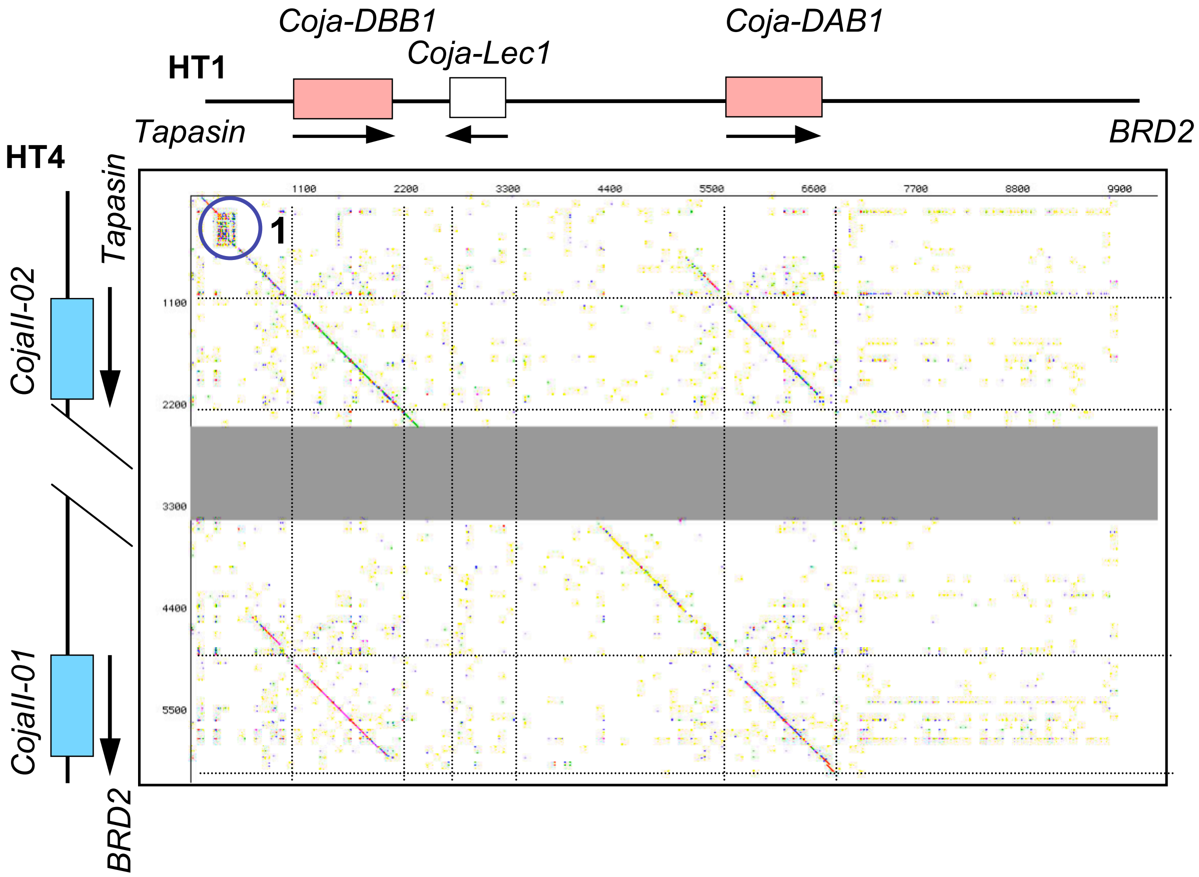

(D) HT1 vs HT5

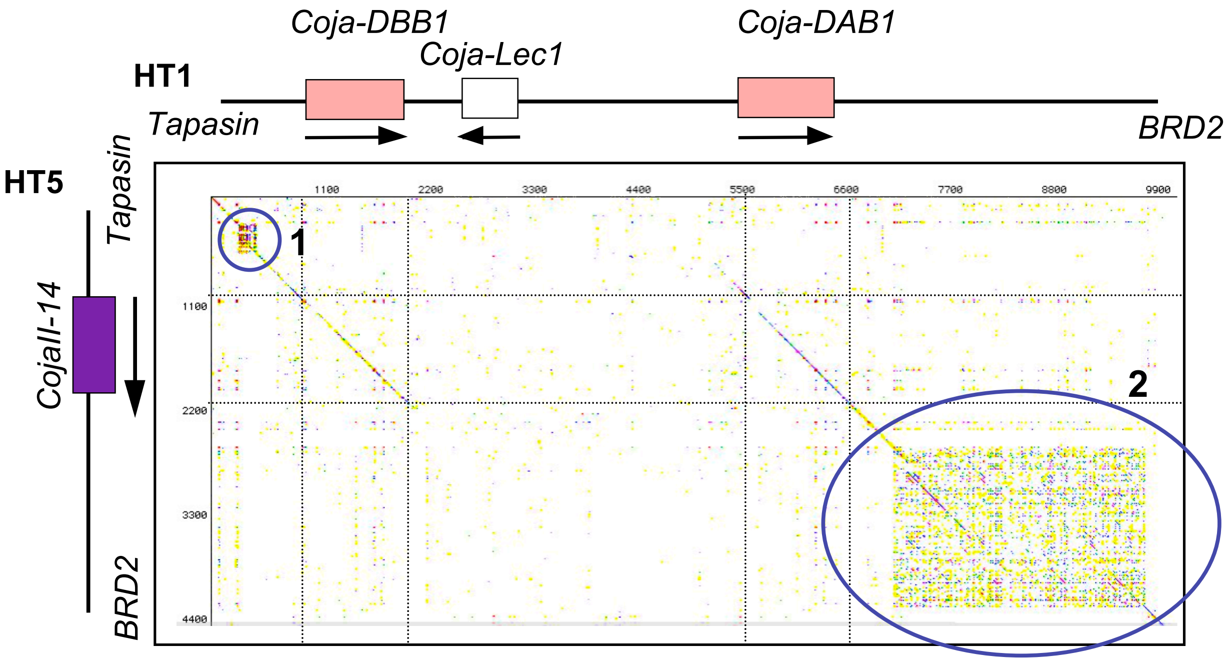

(E) HT2 vs HT3

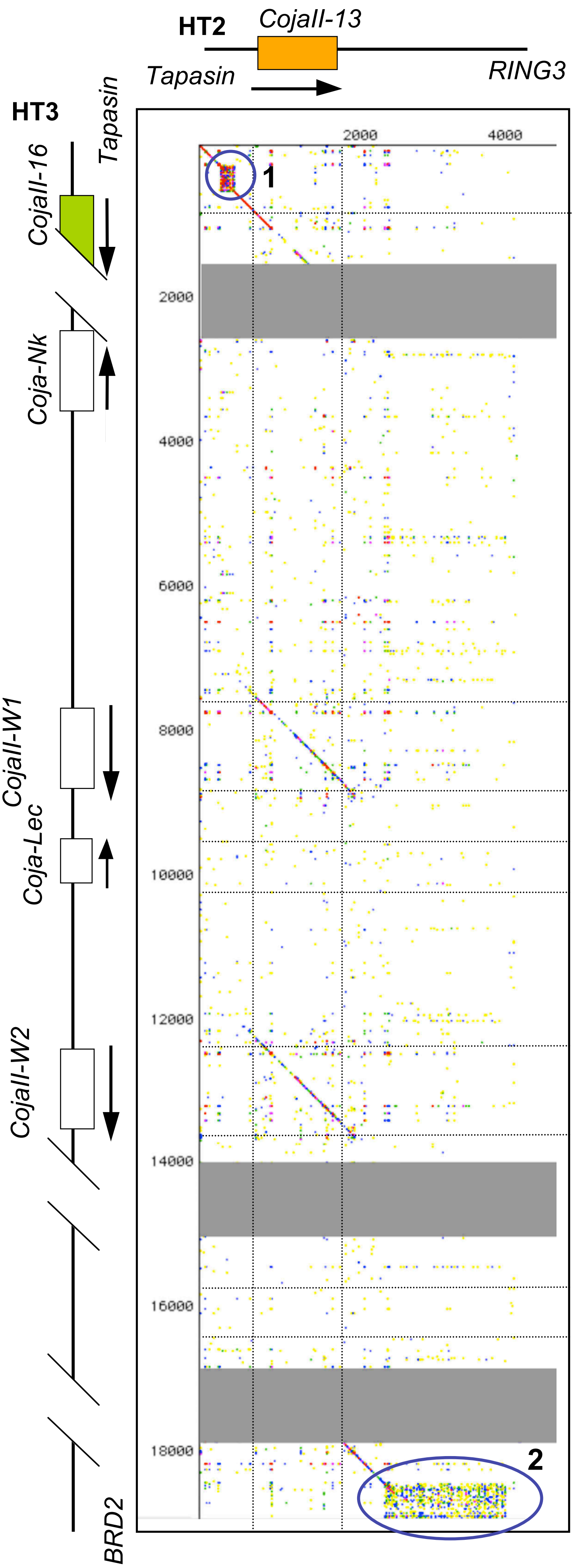
HT2
CojaII-13
Tapasin
RING3
HT3
Tapasin
CojaII-16
Coja-Nk
CojaII-W1
Coja-Lec
CojaII-W2
BRD2
2000
4000
2000
4000
6000
8000
10000
12000
14000
16000
18000
1
2

(F) HT2 vs HT4

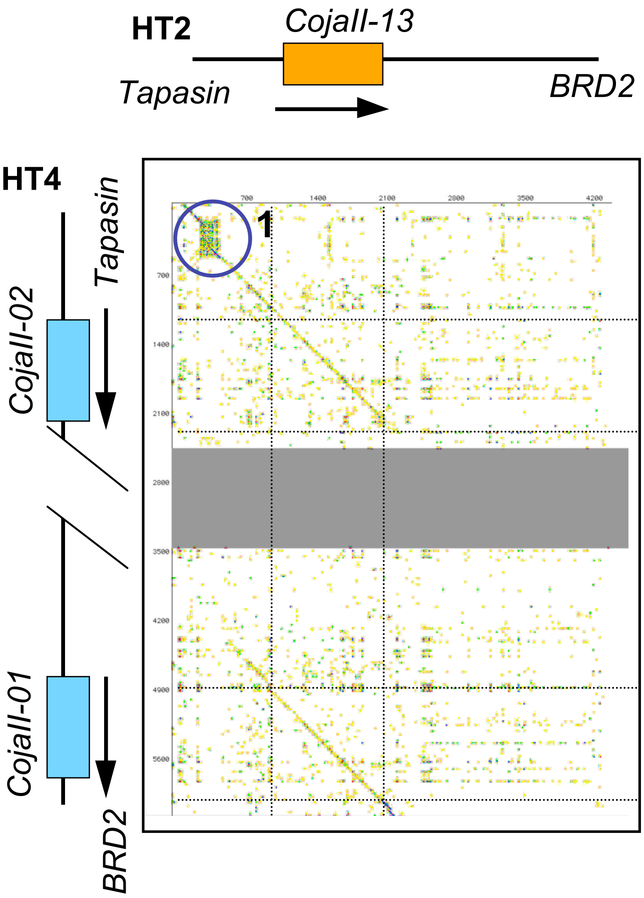

(G) HT2 vs HT5

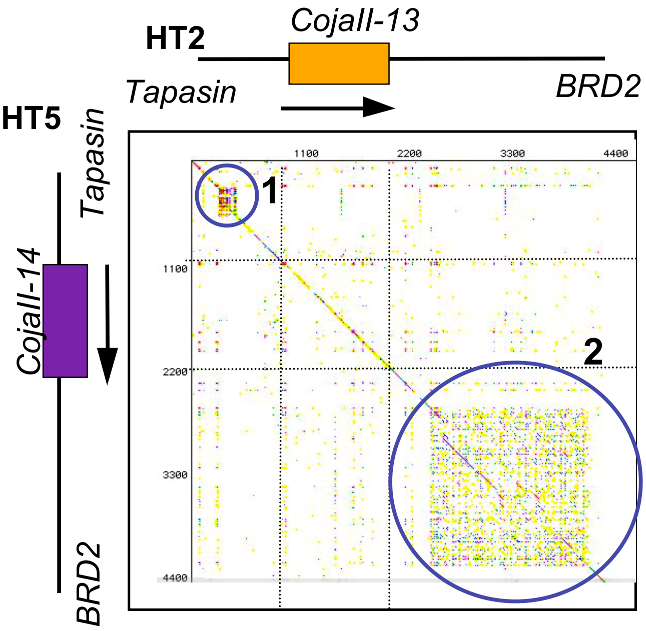

(H) HT3 vs HT4

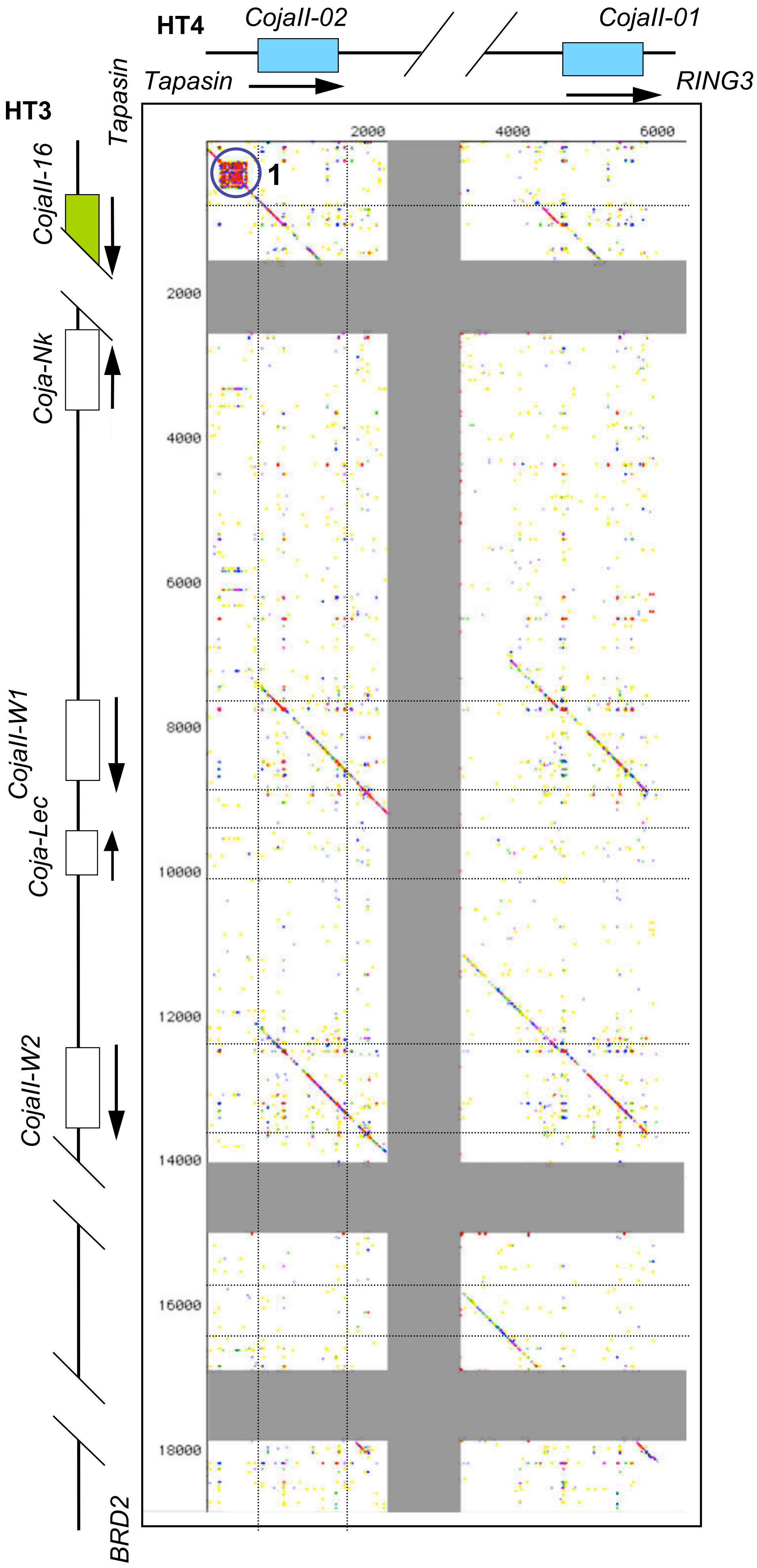

HT4
CojaII-02
CojaII-01
Tapasin
RING3
HT3
Tapasin
CojaII-16
Coja-Nk
CojaII-W1
Coja-Lec
CojaII-W2
BRD2
2000
4000
6000
2000
4000
6000
8000
10000
12000
14000
16000
18000
1

(I) HT3 vs HT5

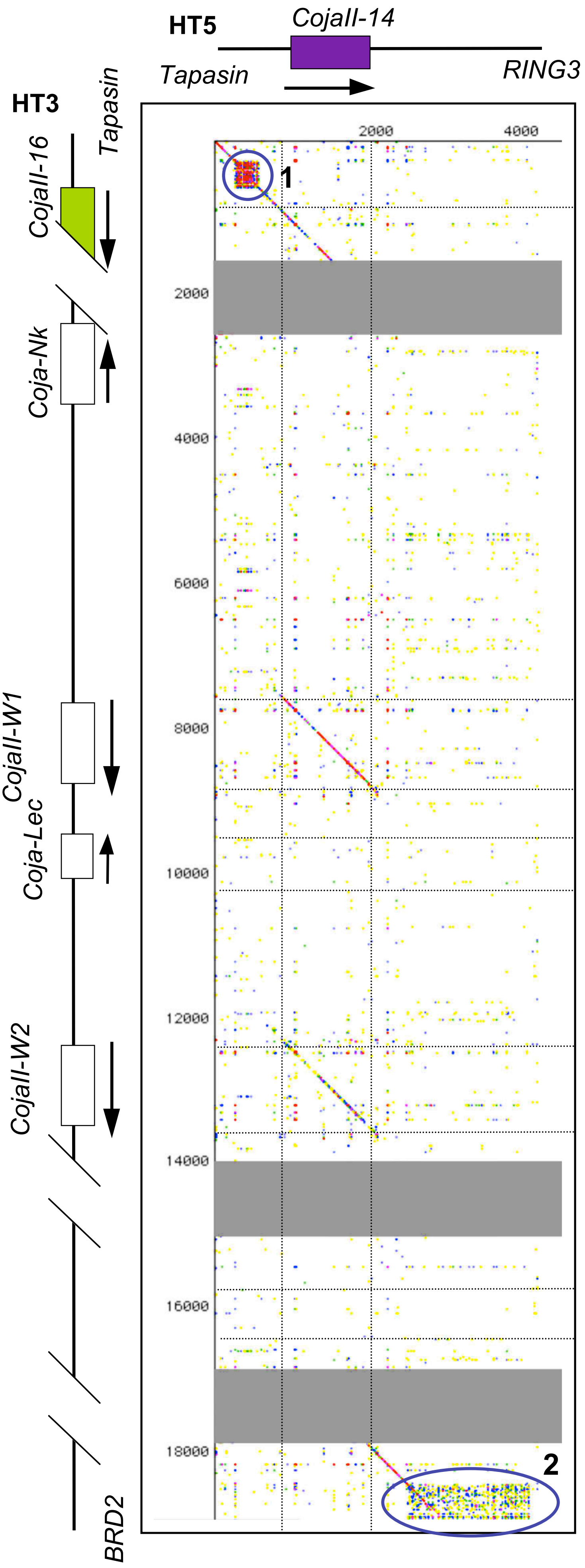

HT5
CojaII-14
Tapasin
RING3
HT3
Tapasin
CojaII-16
Coja-Nk
CojaII-W1
Coja-Lec
CojaII-W2
BRD2
2000
4000
2000
4000
6000
8000
10000
12000
14000
16000
18000
1
2

(J) HT4 vs HT5

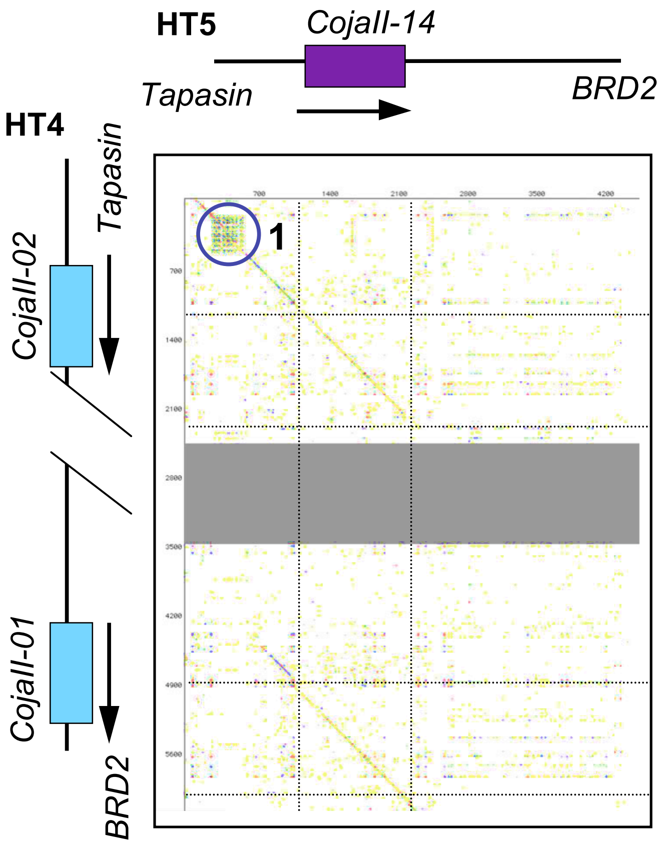
HT5
CojaII-14
Tapasin
BRD2
HT4
Tapasin
CojaII-02
CojaII-01
BRD2
1

(K) HT2 vs B12

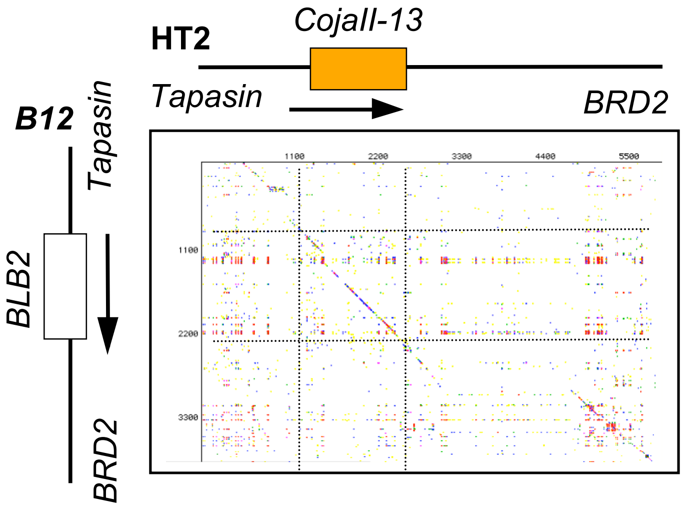

(L) B12 vs B21

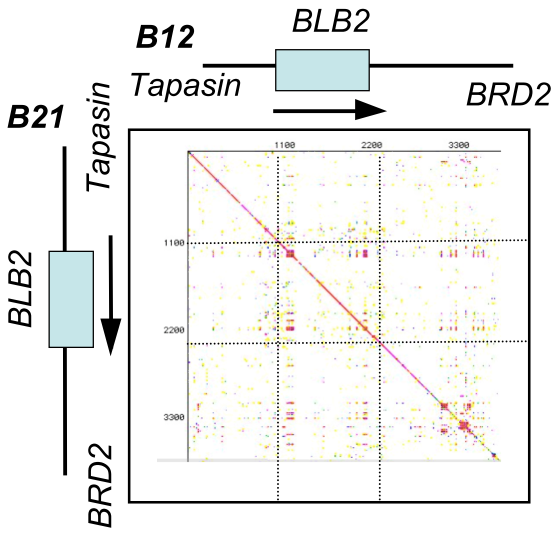

Supplement: Additional file 2 — Dot-matrix analysis among five Coja haplotypes (A ~ J) between quail and chicken (K) and between chicken B12 and B21 (L). Dot plot comparisons shows HT1 vs HT2 (A) HT1 vs HT3 (B) HT1 vs HT4 (C) HT1 vs HT5 (D) HT2 vs HT3 (E) HT2 vs HT4 (F) HT2 vs HT5 (G) HT3 vs HT4 (H) HT3 vs HT5(I) HT4 vs HT5 (J) HT2 vs chicken B12 (K) and chicken B12 vs B21 (L). Numbers and blue circles in the images show the location of genome candidate remodeling (rearrangement) factors with the numbers 1 to 3 representing TB1 – TB3 respectively as outlined in Table 6. Gray background shows the gap within the segments that was determined by sequencing. [file 1471-2164-7-322-S2.pdf]
